# Supplementary material for: DHX15 inhibits mouse APOBEC3 deamination
Source: PLoS Pathog. 2025 Apr 1;21(4):e1013045. doi: 10.1371/journal.ppat.1013045 (PMC11990775; doi:10.1371/journal.ppat.1013045)
Supplement: S1 Table — (PDF) [file ppat.1013045.s001.pdf]

S1 Table. Primers used for cloning

| Name | sequence | RE site | Description |
|---|---|---|---|
| mA3 F1 | AGCCTCGAGatgggaccattctgtct | Xho I | mA3 F1, B1 (full length mAPOCBEC3) |
| mA3 B1 | GTGGAATTCtcaagacatcgggggtcc | EcoR I | |
| mA3 F2 | AGCCTCGAGctgaggcgaatggacccg | Xho I | mA3 F1, B2 (mAPOBEC3 N terminal 199 aa) |
| mA3 B2 | GTGGAATTCtcacattcgcctcagaatctc | EcoR I | mA3 F2, B1 (mAPOBEC3 C terminal 199aa) |
| mA3 B3 | GTGGAATTCataccaggtgatcttgaac | EcoR I | mA3 F1, B3 (mAPOBEC3 N terminal 99 aa) |
| mA3 B5 | GTGGAATTCtcagaagatgtccaggctcag | EcoR I | mA3 F1, B5 (mAPOBEC3 N terminal 127aa) |
| mA3 B8 | GTGGAATTCtcagaaccagtataaaaagcaga | EcoR I | mA3 F1, B8 (mAPOBEC3 N terminal 80 aa) |
| mA3 F4 | AGCCTCGAGgataccttcttgtgctatg | Xho I | mA3 F4, B5 (mAPOBEC3 40-127aa) |
| mA3 F6 | AGCCTCGAGcatggggtctttaagaac | Xho I | mA3 F6, B5(mAPOBEC3 60-127aa) |
| mA3 F8 | AGCCTCGAGgacaaagtactgaaag | Xho I | mA3 F8, B5 (mAPOBEC3 80-127aa) |
| hA3G F1 | AGCCTCGAGatgaagcctcacttcaga | Xho I | hA3G F1, B1 (full length hAPOBEC3G) |
| hA3G B1 | GTGGAATTCtcagttttcctgattctg | EcoR I | |
| hA3G F2 | AGCCTCGAGattctcagacactcgatg | Xho I | hA3G F2, B1 (hAPOBEC3G C terminal 192aa) |
| hA3G B2 | GTGGAATTCTCAaatctcccccagcatgatg | Xho I | hA3GF1, B2 (hAPOBEC3G A3G N terminal 192 aa) |
| hDHX15 F1 | AGCCTCGAGatgtccaagcggcaccgg | Xho I | hDHX15 F1, hDHX15 B1 (human DHX15) |
| hDHX15 B1 | GTGGAATTCtcagtactgtgaatattcc | EcoR I | |
| mDHX15 F1 | AGCCTCGAGatgtccaagaggcatcggtt | Xho I | mDHX15 F1, hDHX15 B1 (mouse DHX15) |
