# Supplementary material for: DHX15 inhibits mouse APOBEC3 deamination
Source: PLoS Pathog. 2025 Apr 1;21(4):e1013045. doi: 10.1371/journal.ppat.1013045 (PMC11990775; doi:10.1371/journal.ppat.1013045)
Supplement: S1 Fig — Lysates were immunoprecipitated with anti-FLAG antibody and probed with anti-GFP (top) or –FLAG (bottom). (PDF) [file ppat.1013045.s002.pdf]

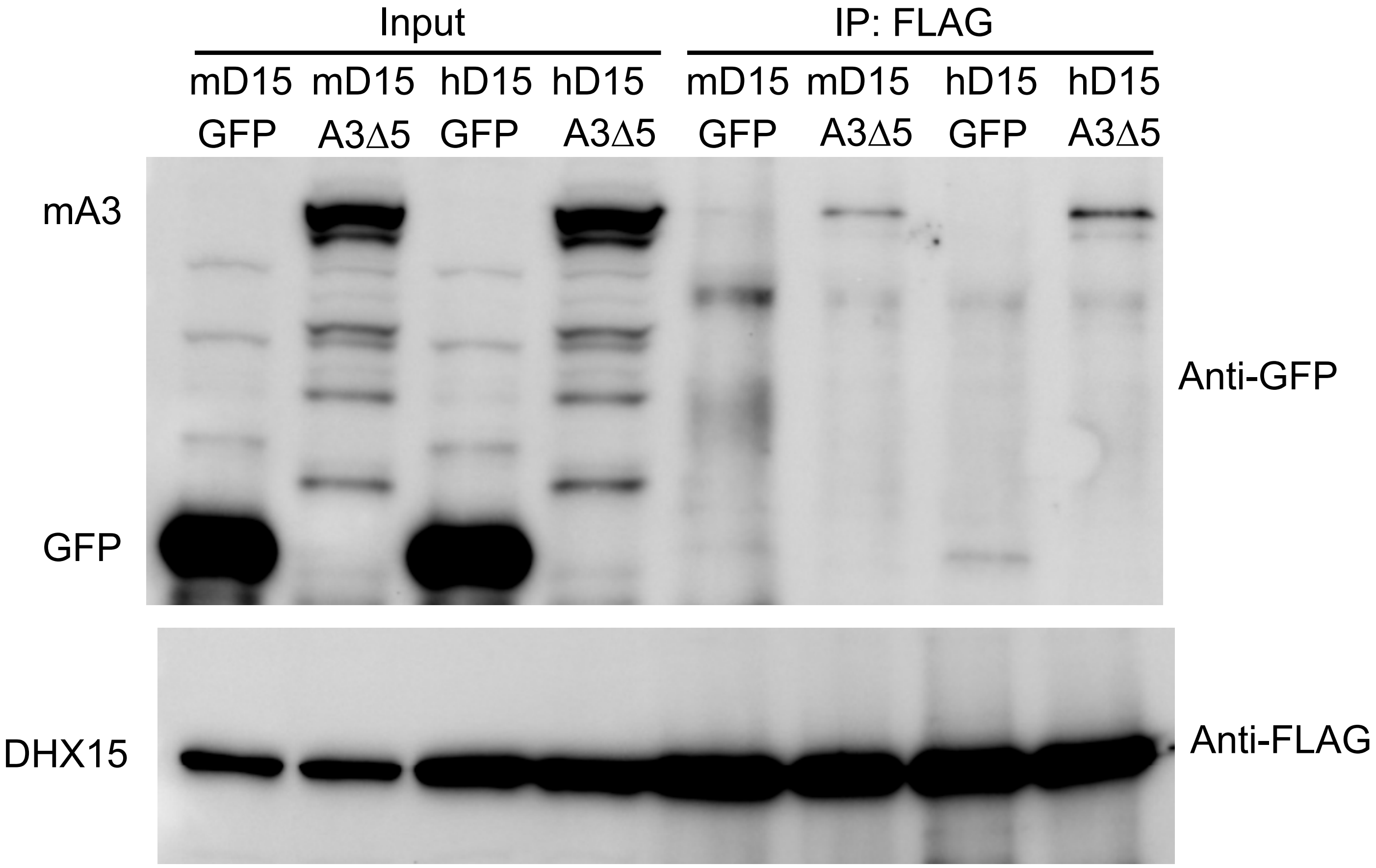

S1 Figure. mAPOBEC3Δ5 binds to both mouse and human DHX15. Co-IP of GFP-tagged mAPOBEC3Δ5 with FLAG-tagged mouse or human DHX15 (D15). Lysates were immunoprecipitated with anti-FLAG antibody and probed with anti-GFP (top) or –FLAG (bottom).
