# Supplementary material for: DHX15 inhibits mouse APOBEC3 deamination
Source: PLoS Pathog. 2025 Apr 1;21(4):e1013045. doi: 10.1371/journal.ppat.1013045 (PMC11990775; doi:10.1371/journal.ppat.1013045)
Supplement: S2 Fig — Cells transfected with HA-tagged mA3 were stained with anti-HA antibody and antibody to endogenously expressed DHX15. (PDF) [file ppat.1013045.s003.pdf]

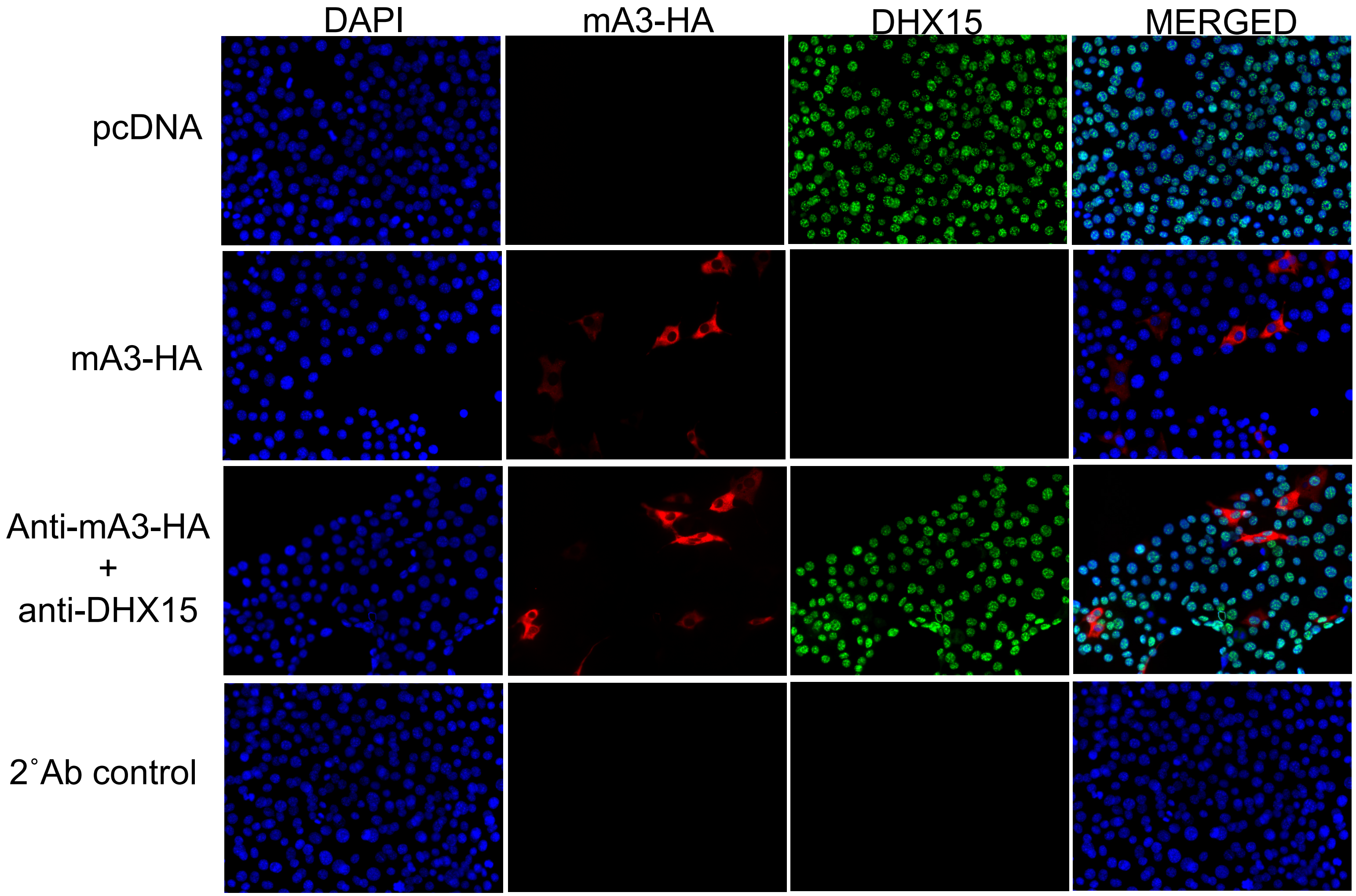

S2 Figure. Expression of transfected mAPOBEC3 in cells used for PLA (see Fig. 1H). Cells transfected with HA-tagged mA3 were stained with anti-HA antibody and antibody to endogenously expressed DHX15.
